# Connectome-based predictive modeling of handwriting and reading using task-evoked and resting-state functional connectivity

**DOI:** 10.1016/j.isci.2025.113075

**Published:** 2025-07-07

**Authors:** Junjun Li, Dai Zhang, Huan Ren, Ke Zhou, Yang Yang

**Affiliations:** 1 State Key Laboratory of Cognitive Science and Mental Health, Institute of Psychology, Chinese Academy of Sciences, Beijing 100101, China; 2 Department of Psychology, University of Chinese Academy of Sciences, Beijing 100049, China; 3 Hangzhou Institute of Medicine, Chinese Academy of Sciences, Hangzhou, Zhejiang 310018, China; 4 Department of Radiology, The Second Affiliated Hospital of Anhui Medical University, Hefei 230601, China; 5 Medical Imaging Research Center, Anhui Medical University, Hefei 230032, China; 6 Beijing Key Laboratory of Applied Experimental Psychology, National Demonstration Center for Experimental Psychology Education (Beijing Normal University), Faculty of Psychology, Beijing Normal University, Beijing 100875, China

**Keywords:** Neuroscience, Cognitive neuroscience

## Abstract

Previous studies have shown that functional connectivity-based models can characterize individual differences in human behavior. However, the applicability of such models to skilled motor behavior remains largely unexplored. In this study, we employed a connectome-based predictive modeling (CPM) approach to predict individual differences in handwriting skills using handwriting task-related and resting-state functional magnetic resonance imaging (fMRI) data. Our results demonstrated that general functional connectivity (GFC) metrics, which capture shared features across task-evoked and resting-state functional connectivity, reliably reflect individual differences in handwriting speed. This predictive model involved multiple functional networks associated with motor, visual, and executive control processes. Furthermore, we found that the GFC-based model derived from handwriting task and resting-state data also predicted individual differences in reading ability, revealing both shared and distinct neural substrates underlying handwriting and reading skills. These findings highlight the potential of neuroimaging in the diagnosis of handwriting- and reading-related disorders.

## Highlights

• CPM using combined task and rest fMRI predicts handwriting ability
• GFC metrics of handwriting speed involve motor, visual, and executive networks
• GFC from handwriting task and rest fMRI predicts reading ability
• Neuroimaging shows potential for diagnosing handwriting and reading disorders

## Graphical abstract

**Figure:**
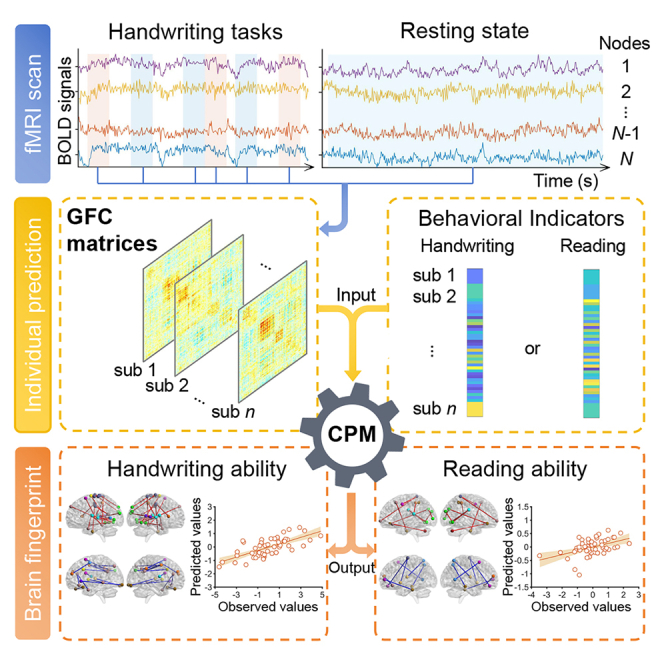

## Introduction

Brain function and structure largely vary across the individuals.^1^^,^^2^^,^^3^ Recently, there has been increasing interest in characterizing the relationship between brain function and structure and behavior at the individual level, offering new insight into the neural basis of human behavior and the clinical application of neuroimaging.^4^^,^^5^^,^^6^ Much effort has been devoted to human cognitive domains, but little is known about skilled motor behavior.

Handwriting is a fundamental and unique visuomotor skill that involves a complex interplay of linguistic, executive control, and motor processes. Despite the widespread use of digital input methods, handwriting remains a crucial component of academic development and professional activities, warranting significant attention.^7^ For example, handwriting difficulties affect 10%–30% of school-age children.^7^ Moreover, handwriting impairments are prevalent in various neurological disorders, including dyslexia,^8^^,^^9^ attention deficit hyperactive disorder,^10^ autism,^11^ developmental coordination disorder,^12^ Parkinson’s disease,^13^ and Alzheimer’s disease.^14^ Almost everyone learns and practices handwriting extensively during school age, resulting in substantial individual differences in handwriting. This has led to the proposal that handwriting features may serve as a critical biometric of health.^15^ For instance, handwriting speed is a typical behavioral index for assessing handwriting skills and exhibits significant individual differences in both the general population^16^^,^^17^ and individuals with neurological disorders.^8^^,^^18^

At the neural level, lesion and noninvasive neuroimaging studies have identified several key regions involved in handwriting processes, including the primary motor cortex, premotor cortex, inferior parietal lobule, superior parietal sulcus, fusiform gyrus, thalamus, and cerebellum,^19^^,^^20^ as well as the interactions between these regions during handwriting.^21^^,^^22^ However, these findings are typically based on population-level analyses, and the neural signatures of individual differences in handwriting skills have not yet been explored.

Connectome-based predictive modeling (CPM) is a powerful tool for developing brain fingerprints of personal traits,^4^^,^^23^ and has been successfully applied to characterize individual differences in general intelligence,^4^ attention,^24^ working memory,^25^ reading,^26^ and personality.^27^ While both task-evoked and resting-state functional connectivity have demonstrated predictive utility for behavior, some studies suggest that task-evoked functional connectivity is more powerful for predicting individual traits.^25^^,^^28^ A recent study has shown that the aggregated features across task-evoked and resting-state functional connectivity—termed general functional connectivity (GFC)—are more reliable and effective for characterizing individual variations in cognitive traits than using task-related or resting-state functional magnetic resonance imaging (fMRI) data alone. This approach is particularly advantageous under limited scanning time, as a minimum of a 20-min scan can achieve good reliability in functional brain networks.^29^

This study aims to identify functional brain networks that account for individual differences in handwriting skill through constructing GFC from handwriting task-related and resting-state fMRI data. We employed handwriting speed as a behavioral variable of handwriting skill for the following reasons. First, handwriting speed can comprehensively reflect the efficiency of handwriting processing and exhibits robust individual differences in both healthy people and those with neurological disorders.^10^^,^^30^^,^^31^ Second, it can be easily and objectively measured, thus holding significance for clinical application. We sought to delineate functional brain networks that signify individual differences in handwriting speed. We hypothesized that the brain fingerprint of handwriting would involve large-scale functional networks, including the frontal-parietal network (FPN), somatosensory motor network (SMN), and visual network (VN).

Finally, we examined whether whole-brain GFC derived from handwriting task-related and resting-state fMRI data could predict individual differences in reading ability, given that handwriting has been shown to be closely related to reading development.^32^^,^^33^ Furthermore, convergent evidence from behavior and neuroimaging studies revealed that handwriting deficits are commonly observed in individuals with reading difficulties.^9^^,^^34^ Accordingly, we investigated the overlapping and distinct functional networks that contribute to the individual differences in handwriting and reading skills, exploring the possibility that handwriting-based brain fingerprints could serve as potential indicators of developmental dyslexia.

## Results

### Behavioral results

**Figure 1:**
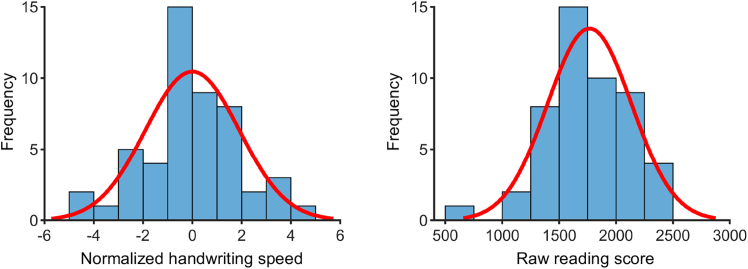
Distribution of the normalized handwriting speed and the raw reading score

This study recruited a total of 56 adult participants (28 males, aged 19–28 years). Six participants were excluded from the data analysis due to excessive head motion during either the task scan or the resting-state scan. For the final sample of 50 participants (23 males, aged 19–28 years), the mean (standard deviation [SD]) time taken to copy high-frequency Chinese characters (HFCs) and low-frequency Chinese characters (LFCs) was 35.10 s (6.33 s) and 40.09 s (7.75 s) in the handwriting speed test. The mean (SD) reading score was 1,766.77 (369.92) evaluated by the character recognition test. Shapiro-Wilk tests revealed that both the normalized handwriting speed (*W* = 0.99, *p* = 0.953) and the raw reading score (*W* = 0.99, *p* = 0.752) were normally distributed (Figure 1).

### Prediction performance of handwriting speed

**Figure 2:**
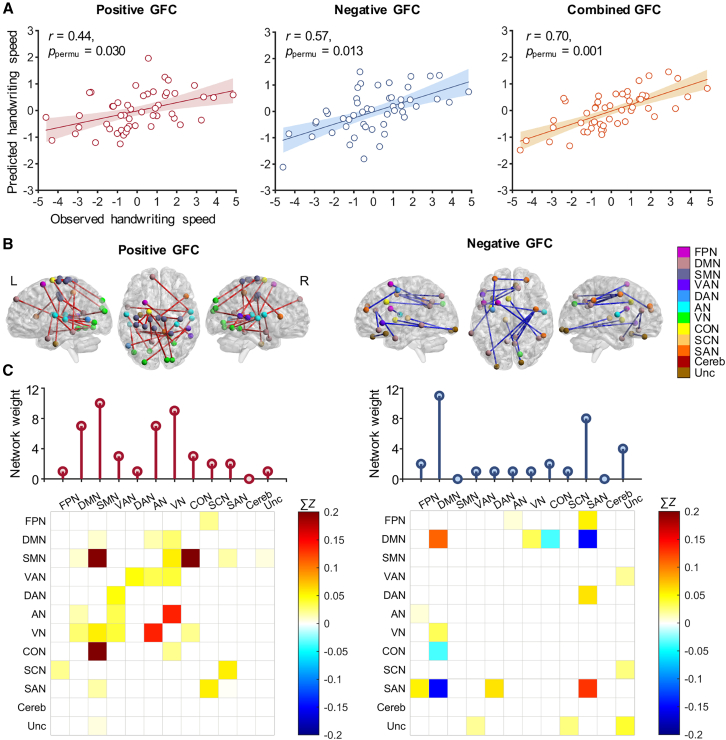
Predicting handwriting speed based on GFC sharing features of handwriting task-related and resting-state fMRI data (A) Scatterplots showing the correlations between observed scores and predicted scores of handwriting speed based on positive, negative, and combined (positive and negative) GFC. (B) Positive and negative GFC contributing to handwriting speed predictions. The colors of the nodes indicate the networks to which they belong. (C) Network distribution of the predictive GFC and the weight of each network. Network weights are calculated as the sum of node degrees within that network. Matrix plots represent the connectivity strength between pairs of the 12 brain networks. The colorbars map the color of each matrix element to the sum of the connectivity strength (Fisher’s *Z* scores) across all the edges connecting the networks. GFC, general functional connectivity; FPN, frontal-parietal network; DMN, default mode network; SMN, somatosensory motor network; VAN, ventral attention network; DAN, dorsal attention network; AN, auditory network; VN, visual network; CON, cingulo-opercular network; SCN, subcortical network; SAN, salience network; Cereb, cerebellum; Unc, uncertain; L, left; R, right. *p*_permu_ = the *p* value obtained by the permutation test.

The CPM models successfully predicted individual handwriting speed using the positive GFC (*r* = 0.44, the *p* value obtained by the permutation test: *p*_permu_ = 0.030), negative GFC (*r* = 0.57, *p*_permu_ = 0.013), as well as the combined GFC (*r* = 0.70, *p*_permu_ = 0.001) (Figure 2A). The positive GFC that appeared at least 90% of the iterations included 23 edges, representing 0.066% of total number of edges (Figure 2B), predominantly contributed by the SMN (including positive intra-network connectivity within the SMN, and positive inter-network connectivity between the SMN and VN/cingulo-opercular network [CON]) and the VN (including the positive inter-network connectivity between the VN and auditory network [AN]/SMN/ventral attention network [VAN]/default mode network [DMN]) (Figure 2C). The negative GFC included 16 edges, representing 0.046% of total number of edges (Figure 2B), with the majority being attributed to the DMN (including positive intra-network connectivity within the DMN, the positive inter-network connectivity between the DMN and VN, and negative inter-network connectivity between the DMN and salience network [SAN]/CON) and the SAN (including the positive intra-network connectivity within the SAN, the positive inter-network connectivity between the SAN and FPN/dorsal attention network [DAN], and the negative inter-network connectivity between the SAN and DMN) (Figure 2C).

### Internal validation results

Using a more lenient threshold (*p* < 0.0025), CPM models continued to predict handwriting speed with positive (*r* = 0.39, *p*_permu_ = 0.046), negative (*r* = 0.54, *p*_permu_ = 0.004), and combined GFC (*r* = 0.67, *p*_permu_ = 0.001) (see Figure S1). When a more stringent threshold (*p* < 0.0005) was applied, the predictive performance using positive GFC alone did not reach statistical significance (*r* = 0.37, *p*_permu_ = 0.116), but models using negative GFC (*r* = 0.47, *p*_permu_ = 0.038) and combined GFC (*r* = 0.59, *p*_permu_ = 0.015) remained significant (see Figure S2). When the time series of both the task and resting states were band-pass filtered by 0.008–0.09 Hz, predictions using negative (*r* = 0.61, *p*_permu_ = 0.002) and combined GFC (*r* = 0.57, *p*_permu_ = 0.007) remained significant, whereas positive GFC did not (*r* = 0.21, *p*_permu_ = 0.243) (see Figure S3). Moreover, consistent with the main results, the validation analyses demonstrated that positive GFC are dominated by the SMN and the VN, and negative GFC were dominated by the DMN and the SAN.

Finally, when utilizing the Craddock atlas for node definition, the combined CPM model was still able to accurately predict handwriting speed (positive: *r* = 0.33, *p*_permu_ = 0.097; negative: *r* = 0.26, *p*_permu_ = 0.174; combined: *r* = 0.42, *p*_permu_ = 0.031) (see Figure S4). Overall, these results demonstrated that handwriting speed can be reliably predicted by the GFC constructed by integrating task-related and resting-state features, with the combined CPM model being the most robust.

Sensitivity analyses indicated that when different thresholds were applied to identify and visualize the GFC stably related to handwriting speed across the CPM cross-validation procedure, there was no significant change in the network distribution patterns of the resulting features. Specifically, at a threshold of 85%, the number of positive edges was *n*_pos_ = 26, the number of negative edges was *n*_neg_ = 18, and the correlation coefficient of network connectivity matrices were *r*_pos_ ≈ 1, *p*_pos_ < 0.001, and *r*_neg_ ≈ 1, *p*_neg_ < 0.001. At a 95% threshold, *n*_pos_ = 18, *n*_neg_ = 11, *r*_pos_ = 0.90, *p*_pos_ < 0.001, *r*_neg_ = 0.83, and *p*_neg_ < 0.001. At a 100% threshold, *n*_pos_ = 7, *n*_neg_ = 7, *r*_pos_ = 0.57, *p*_pos_ < 0.001, *r*_neg_ = 0.76, and *p*_neg_ < 0.001 (see Figure S5).

### External validation results

A dataset of 24 children (11 males, mean ± SD age = 11.09 ± 0.83 years) was used for external validation. The mean (SD) handwriting speed was 61.71 (9.32) characters per minute. CPM analysis revealed significant prediction of handwriting speed using negative GFC (*r* = 0.54, *p*_permu_ = 0.040) and combined GFC (*r* = 0.53, *p*_permu_ = 0.045) (see Figure S6). These findings collectively demonstrated that the predictive models generalize across populations with varying developmental levels of handwriting and reading abilities.

### GFC model based on handwriting and resting-state data predicts reading ability

**Figure 3:**
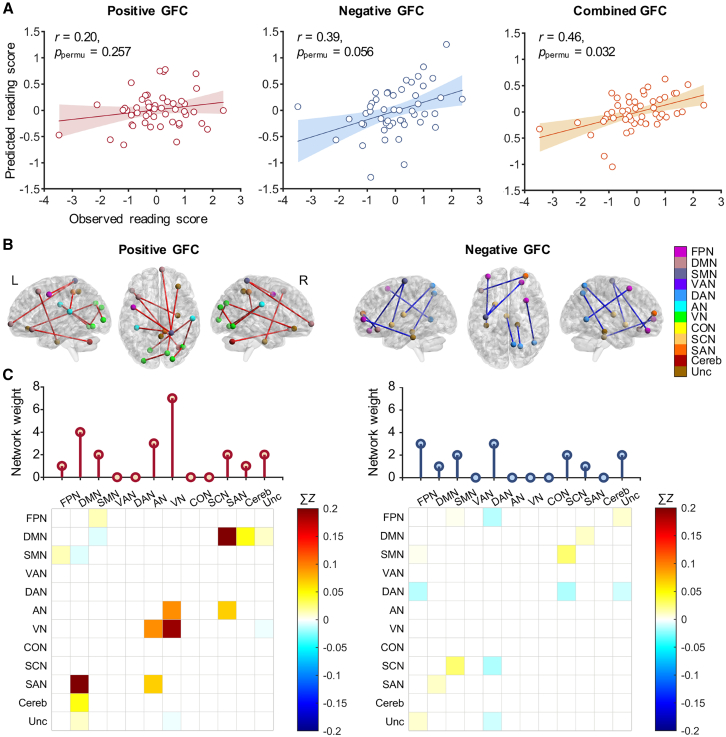
Predicting reading scores based on GFC sharing features of handwriting task-related and resting-state fMRI data (A) Scatterplots showing the correlations between observed reading scores and predicted reading scores based on positive, negative, and combined GFC. (B) Positive and negative GFC contributing to the predictions of reading score. (C) Network distribution of the predictive GFC and the weight of each network. Network weights are calculated as the sum of node degrees within that network. Matrix plots represent the connectivity strength between pairs of the 12 brain networks. The colorbars map the color of each matrix element to the sum of the connectivity strength (Fisher's *Z* scores) across all edges connecting the networks. GFC, general functional connectivity; FPN, frontal-parietal network; DMN, default mode network; SMN, somatosensory motor network; VAN, ventral attention network; DAN, dorsal attention network; AN, auditory network; VN, visual network; CON, cingulo-opercular network; SCN, subcortical network; SAN, salience network; Cereb, cerebellum; Unc, uncertain; L, left; R, right. *p*_permu_ = the *p* value obtained by the permutation test.

Using a threshold of *p* < 0.001 to select GFC that was correlated with reading scores, CPM analysis revealed that the combined model accurately predicted reading ability (positive: *r* = 0.20, *p*_permu_ = 0.257; negative: *r* = 0.39, *p*_permu_ = 0.056; combined: *r* = 0.46, *p*_permu_ = 0.032) (Figure 3A). The positive GFC (occurring > 90% of the iterations) included 11 edges (0.032% of total number of edges), mainly contributed by the DMN and the VN, and the negative GFC included 7 edges (0.020% of total number of edges), mainly contributed by the FPN and the DAN (Figures 3B and 3C).

## Discussion

In this study, we utilized the CPM approach to characterize individual differences in handwriting speed based on the shared features across task-evoked and resting-state functional brain connectivity. Our predictive models revealed that a large-scale functional network encompassing the SMN, VN, DMN, SAN, and AN contributes significantly to behavioral performance, suggesting that individual variations in handwriting skill are reflected by distributed brain systems. Additionally, we found that the GFC derived from handwriting task-related and resting-state fMRI data could also predict reading skills, providing new evidence for a brain-based link between handwriting and reading. Collectively, our findings suggest that individual differences in handwriting and reading can be effectively captured through combined measures of task-related and resting-state functional connectivity, highlighting the potential application of neuroimaging in evaluating handwriting- and reading-related disorders.

CPM has been validated as a reliable approach for building predictive models of individual differences in cognition based on task-related or resting-state data.^23^^,^^24^^,^^25^^,^^28^ Consistent with existing literature, we found that CPM could predict individual differences in handwriting, suggesting the feasibility of using CPM in the domain of complex skilled functions. Although handwriting has been recognized as a potential biometric marker of physical and mental health,^15^ there remains a gap in characterizing individual differences in handwriting at the neural level. Our findings provide new insights into this area through the application of multimodal neuroimaging measures. Moreover, we found that for both handwriting and reading, the GFC metrics derived from shared features of task and resting states achieved high prediction accuracy. Task-related and resting-state data each have unique advantages in data collection, model construction, and behavioral correlation. Thus, combining them provides a powerful strategy to maximize their strengths and minimize the limitations of either modality.^29^ Our results support the claim that constructing GFC by integrating task-related and resting-state data may be a promising approach for building predictive models across different functional domains.^29^

Specifically, we found that the GFC positively associated with handwriting speed was primarily attributed to the SMN and VN, including both the intra-network connectivity within the SMN and the inter-network connectivity between the SMN and VN. These networks have previously been implicated in handwriting processing in group-level studies.^22^^,^^35^ Our findings extend this understanding by suggesting that the neural efficiency of task-relevant brain networks plays a critical role in explaining individual differences in handwriting speed. Evidence from motor learning and development studies has shown that motor-skill learning is associated with increased functional integration of motor circuits.^36^^,^^37^ Similarly, increased regional activation in visual regions during visuomotor learning has been linked to improved response time.^38^ Based on these findings, we hypothesize that faster handwriting speed is derived from the specialization of motor and visual circuits and the enhanced integration and cooperation between them. Furthermore, we found that the networks that facilitated fast handwriting speed included inter-network connectivity between the SMN and CON. The CON, an attentional control network, functions as a control hub over other brain networks, initiating goal-directed behaviors and maintaining executive control in relation to task objectives. The connectivity between the CON and sensorimotor regions has been shown to increase with upper-limb disuse, supporting specific control over motor execution.^39^ Thus, the connectivity between the CON and SMN likely represents top-down executive regulation of low-level sensorimotor processes, facilitating faster handwriting speed.

Conversely, handwriting speed was negatively predicted by positive intra-network connectivity within the DMN and SAN, as well as by negative inter-network connectivity between DMN and SAN. The DMN is a core network whose activity typically decreases during task engagement and attentional states, although its exact function remains controversial. Traditionally, the DMN is thought to support self-referential processes, such as autobiographical memory and mind wandering.^40^^,^^41^ However, emerging evidence also suggests that the DMN may play a role in cognitive processing^42^ and motor execution.^43^^,^^44^ Our findings align with the view that the DMN supports automated information processing.^45^ The SAN, which includes nodes in the cingulate cortex, supramarginal gyrus and rostral prefrontal cortex, functions as a detector for external stimuli that demand immediate attention.^46^ Increased connectivity within the DMN and SAN may reflect the intrinsic characteristics of these networks, as well as greater cognitive effort required for handwriting control due to reduced automation. During cognitively demanding tasks, the SAN typically shows increased activation, while the DMN is suppressed, reflecting a functional segregation between the two networks.^47^^,^^48^ This segregation between SAN and DMN is thought to facilitate cognitive control.^48^^,^^49^ Thus, greater negative connectivity between the SAN and DMN may benefit handwriting speed, as it allows for the inhibition of task-irrelevant brain activity and enhances processing efficiency. In contrast, individuals with stronger positive connectivity between the SAN and DMN tend to exhibit slower handwriting speed, possibly due to the increased involvement of the SAN in task-unrelated processes, such as mind wandering or self-referential processing governed by the DMN. This redirection of attentional resources may disrupt motor coordination and increase the cognitive load required to maintain focus, thereby slowing handwriting speed. This underscores the importance of balanced interactions between large-scale brain networks in supporting efficient skilled behavior.

Finally, we found that whole-brain GFC, constructed from both handwriting task-related and resting-state data, successfully predicts individual differences in reading performance. To our knowledge, this is the first instance of establishing a correspondence between handwriting and reading at the individual level using brain-based modeling approach. The relationship between handwriting and reading has been extensively explored across various writing systems.^32^^,^^50^^,^^51^^,^^52^ It has been proposed that handwriting serves as foundational scaffolding for reading development by reinforcing motor memory of characters and refining orthographic representations.^32^^,^^53^ Our findings provide additional neuroimaging evidence to support and expand this view. Furthermore, handwriting deficits have been identified as a salient symptom of dyslexia, and handwriting assessments are often used in its diagnosis.^9^^,^^34^^,^^54^ Within the handwriting-based networks, we identified that the intra-network connectivity within the VN, and the inter-network connectivity between the VN and AN contributed most significantly to the prediction of reading ability. This is consistent with the understanding that visual orthographic processing underlies both handwriting and reading. It has been suggested that handwriting can promote reading development by strengthening orthographic representations^32^ or by increasing sensitivity to visual variations.^55^ However, at the edge level, no overlapping edges were found between the networks that robustly predict handwriting speed and those that predict reading performance, suggesting that while both skills share visual orthographic processing mechanisms, they may rely on distinct neural pathways. In handwriting, the VN connects with multiple other networks, including the SMN and VAN, to support the motor output of graphemes.^22^ In contrast, reading is primarily supported by the connectivity within the VN and between the VN and AN, reflecting processes involved in grapheme analysis and the grapheme-to-phoneme conversion. These findings suggest that future research should further investigate the neural relationships between handwriting and reading through the lens of large-scale functional brain networks.

In conclusion, this study demonstrated that handwriting speed can be characterized by GFC derived from shared features of task-related and resting-state fMRI data. Multiple functional brain networks contributed to the predictive models, suggesting that handwriting is a complex skill that integrates cognitive and motor processes across distributed neural systems. Furthermore, we found that the GFC model integrating handwriting task and resting-state data successfully predicted individual differences in reading, providing evidence for a strong connection between handwriting and reading from the perspective of individual differences. These findings suggest that skilled behavior, such as handwriting and reading, can be objectively assessed using neuroimaging, offering novel insights for quantifying the development of handwriting and reading and diagnosing related disorders.

### Limitations of the study

This study has several limitations. First, the relatively small sample size may constrain the generalizability of our findings. A larger and more diverse cohort would provide a more robust basis for validating the results and ensuring their applicability across broader populations. Additionally, due to this small sample size, we could not directly assess sex-based differences in brain fingerprints associated with handwriting and reading abilities—a gap that could be addressed in future research with more extensive sampling. Third, our predictive models were built based on Chinese handwriting, which differs significantly from alphabetic scripts in both visual and motor aspects. Prior studies have highlighted discrepancies in cognitive processes and neural underpinnings between Chinese and alphabetic languages.^32^^,^^56^^,^^57^^,^^58^ As such, it remains to be explored whether and to what extent our predictive models can be applied to predict handwriting and reading in alphabetic languages, which would enhance our understanding of the unique and shared brain fingerprints of handwriting and reading abilities across different cultural backgrounds.

## Resource availability

### Lead contact

Any information required to reanalyze the data reported in this paper is available from the lead contact, Yang Yang (yangyang@psych.ac.cn).

### Materials availability

This study did not generate new materials.

### Data and code availability

• The fMRI data used in this study cannot be deposited in a public repository, as this would conflict with the consent signed by participants. Therefore, any requests for data must be submitted to the lead contact.
• The fMRI data were processed with SPM12. Functional connectivity matrices were constructed using the CONN Functional Connectivity Toolbox. The GFC matrices used for individual behavior prediction, as well as the MATLAB codes used in the CPM analysis have been deposited in the GitHub repository and are publicly available at https://github.com/Junjun-Li2020/PsychCAS-Yang-Lab-CPM as of the date of publication. Accession numbers are listed in the key resources table.
• Any additional information required to reanalyze the data reported in this paper is available from the lead contact upon request.

## Acknowledgments

The authors are grateful to all participants who took part in this study. This work was supported by the 10.13039/501100010007Sino-German Center for Research Promotion (grant number M-0705), the 10.13039/501100001809National Natural Science Foundation of China (grant number 32271120), the 10.13039/501100012166National Key R&D Program of China (grant number 2023YFC3341301), and the Beijing Natural Science Foundation (grant number 5222027).

## Author contributions

J.L., writing – original draft, writing – review and editing, conceptualization, data curation, methodology, and visualization. D.Z., writing – review and editing, methodology, resources, and validation. H.R., Writing – original draft, methodology, data curation, and project administration. K.Z., writing – review and editing, conceptualization, methodology, resources, supervision, and validation. Y.Y., writing – original draft, writing – review and editing, funding acquisition, conceptualization, methodology, supervision, and validation. All authors read and approved the final manuscript.

## Declaration of interests

The authors declare no competing interests.

## STAR★Methods

### Key resources table

**Table:** 

REAGENT or RESOURCE	SOURCE	IDENTIFIER
**Software and algorithms**		

MATLAB R2020a	MathWorks	https://www.mathworks.com/
SPM12 (7219)	Wellcome Department of Cognitive Neurology, University College London, London	https://www.fil.ion.ucl.ac.uk/spm/software/spm12/
CONN Functional Connectivity Toolbox 20.b	the Computational Neuroscience Research Lab, headed by Alfonso Nieto-Castanon	https://web.conn-toolbox.org/
Connectome-based predictive modeling (CPM)	Shen et al.^23^	N/A
BrainNet Viewer v1.62	Xia et al.^59^	https://www.nitrc.org/projects/bnv/
IBM SPSS Statistics 25.0	IBM	https://www.ibm.com/spss?lot=2&mhsrc=ibmsearch_a&mhq=ibm%20spss%20software
Matlab codes used in the CPM analysis	Github	https://github.com/Junjun-Li2020/PsychCAS-Yang-Lab-CPM

### Experimental model and study participant details

#### Participants

A total of 56 adult participants (28 males, aged 19–28 years, mean ± SD age = 22.36 ± 2.32 years) were recruited for the main analysis of this study from universities and research institutions in Beijing, China. Additionally, a group of 24 children (11 males, aged 9–13 years, mean ± SD age = 11.09 ± 0.83 years) were recruited for the external validation of the predictive model from primary schools in Beijing, China. All participants self-identified as Asian, and were native Mandarin speakers. They were right-handed as assessed by a handedness inventory.^60^ Ancestry and ethnicity were not directly collected; however, given the recruitment context and linguistic background, the samples were presumed to primarily consist of individuals with Han Chinese cultural roots. Participants had normal hearing and normal or corrected-to-normal vision, and reported no history of neurological disease or psychiatric disorder.

#### Ethics approval statement

Ethical approval was obtained from the Ethics Committee of the Institute of Psychology, Chinese Academy of Sciences, and the experiments were conducted in accordance with the approved guidelines. Free and informed consent was obtained from each adult participant and each child's guardian prior to the experiment.

### Method details

#### Handwriting and reading tests

All adult participants completed a handwriting speed test and a reading ability test. Handwriting speed was evaluated using a copying task, in which participants were required to copy 20 HFCs and 20 LFCs as fast as possible, respectively. The time taken to copy HFCs/LFCs was recorded. The inverse of the time was calculated and then normalize using a *Z*-score to measure handwriting speed. The sum of the *Z*-scores for HFCs and LFCs represented each participant’s handwriting speed.

Reading ability was measured using a Chinese character recognition test, in which 179 single Chinese characters were presented and participants were asked to compose meaningful words or phrases using the target characters. Each correct response was awarded one point, with the final reading score being calculated based on the norm from a cohort of 100 college students.^61^

#### Stimuli and task procedure

The adult participants performed a delayed copying task during the fMRI scan. The stimuli consisted of 60 single Chinese characters, including 30 HFCs (mean frequency = 1009 times per million, mean number of strokes = 6) and 30 LFCs (mean frequency = 0.98 times per million, mean number of strokes = 6). Participants were instructed to write each character in a stroke-by-stroke manner while minimizing movement of their upper arm and forearm (thereby reducing potential head movement artifacts in the fMRI data). During copying, participants received immediate visual feedback (“ink”) to approximate real handwriting conditions.

Each participant completed one run at natural speed and one run at fast speed. For the natural speed condition, participants were instructed to write at the pace they used in daily life. For the fast speed condition, they were instructed to write as fast as possible while maintaining legible writing. The order of the two runs was counterbalanced across participants. The attributes of the stimuli, including word frequency and stroke numbers, were matched between the natural and fast conditions.

A block design was used in the fMRI experiment, with each run consisting of three blocks of copying HFCs, three blocks of copying LFCs and three blocks of drawing symbols (used as the control condition for brain activation and therefore not analyzed in this study), presented in a pseudorandom order. Each block comprises five trials. In each trial, a fixation (“+”) was first presented centrally for 0.3 seconds. Then, a character was presented for 1 second, followed by a 4.7-second response period. Four blocks of central fixation, each lasting 12 seconds, were interspersed among the task blocks in each run. The total duration of each run was 318 seconds.

Handwriting data were captured using a tablet system specifically developed for fMRI experiments. The tablet system was equipped with a touch-sensitive surface, a force-sensitive stylus, and an adjustable support frame, which was designed to maintain MRI compatibility without adversely affecting fMRI data quality.^62^ The support frame was meticulously adjusted for each participant to ensure a comfortable handwriting experience throughout the imaging session and to allow tablet interaction with the forearm or wrist resting on the support, thereby preventing handwriting fatigue due to gravity.

Additionally, each participant completed a resting-state scan. During this scan, participants were required to stay awake with their eyes closed. The total duration of resting-state scan was 480 seconds.

#### MRI data acquisition

MRI data were acquired using a 3 T MRI system (MAGNETOM Prisma^fit^, Siemens, Erlangen, Germany) at the Beijing MRI Center for Brain Research of the Chinese Academy of Sciences. High spatial resolution structural images were acquired using a three-dimensional T1-weighted, magnetization-prepared rapid acquisition gradient echo (MPRAGE) sequence (repetition time [TR] = 2200 ms, echo time [TE] = 2.08 ms, slice thickness = 1 mm, in-plane resolution = 1.0 mm × 1.0 mm, flip angle [θ] = 8°). Blood oxygenation level-dependent (BOLD) functional MRI data were acquired during both the delayed copying task and the resting state using a gradient-echo echo planar imaging (EPI) sequence^63^ (TR = 1000 ms, TE = 30 ms, slice thickness = 2.2 mm, in-plane resolution = 2.2 mm × 2.2 mm, θ = 45° and 64 axial slices).

#### fMRI data preprocessing

fMRI preprocessing was carried out using the SPM12 freeware (http://www.fil.ion.ucl.ac.uk/spm/, Wellcome Department of Cognitive Neurology, University College London, London). The time series data were first corrected for slice timing and head motion (the first 10 volumes were discarded before slice timing for resting-state fMRI data). The corrected images were coregistered to the corresponding anatomical imaging. The anatomical images were then normalized to Montreal Neurological Institute (MNI) stereotactic space. The derived transformation parameters were applied to normalize the fMRI time series to MNI space using cubic voxels, at a spatial resolution of 2 mm × 2 mm × 2 mm. Finally, the fMRI images were spatially smoothed using an isotropic Gaussian kernel with a full-width at half-maximum (FWHM) of 6 mm.

One adult participant was excluded from the data analysis due to excessive head motion (>3 mm translation or >3° rotation) during the task scan, and five others were excluded due to excessive head motion (>3 mm translation or >3° rotation) during the resting-state scan. Therefore, the final sample included 50 adult participants. In addition, average framewise displacement (FD) was estimated for each participant based on the six head motion parameters.^64^ Among the 50 participants included in the following analysis, the mean (SD) of the average FD was 0.150 mm (0.036 mm) for the natural copying run, 0.152 mm (0.044 mm) for the fast copying run, and 0.134 mm (0.037 mm) for the resting-state run. Only two participants had average FD values that did not meet the stringent threshold of less than 0.25 mm,^65^ but their average FD values did meet the more lenient threshold of less than 0.5 mm.^66^

#### Network construction

The whole-brain connectivity matrices were constructed based on a validated functional parcellation template, which consisted of 264 cortical and subcortical regions represented as 10-mm diameter spheres. These regions were assigned to 11 well-established functional networks, namely the frontal-parietal network (FPN), default mode network (DMN), somatosensory motor network (SMN), ventral attention network (VAN), dorsal attention network (DAN), auditory network (AN), visual network (VN), cingulo-opercular network (CON), subcortical network (SCN), salience network (SAN) and cerebellum.^67^ The BOLD timeseries for each task condition and resting state were estimated using the CONN Functional Connectivity Toolbox.^68^ The effect of nuisance covariates, including fluctuations in BOLD signals from cerebrospinal fluid, white matter and their derivatives, were estimated and removed using the anatomical component correction (CompCor) strategy.^69^ Head motion noise (estimated with six degrees of freedom during the motion correction step) as well as the task effects of each condition convolved with a hemodynamic response function and their first-derivative terms were also regressed out. The resulting residual time series of task conditions were high-pass filtered at 0.008 Hz to preserve task-relevant high-frequency signals,^70^^,^^71^ while that of resting-state scan were band-pass filtered at 0.008–0.09 Hz to reduce low-frequency drift and high-frequency noise effects.^25^ Average timeseries of the 264 regions were extracted independently from each task condition (copying HFCs at natural speed, copying LFCs at natural speed, copying HFCs at fast speed, and copying LFCs at fast speed) and resting-state session, and then concatenated and recombined.^29^ GFC integrating features of handwriting tasks and resting state was derived from these combined timeseries by calculating Pearson's correlation coefficients between all region pairs and transforming them into Fisher's *Z* scores. After that, an undirected weighted 264 × 264 functional connectivity matrix was obtained for each participant, including 34,716 GFC (i.e., features). The GFC metrics demonstrate good test-retest reliability, thereby enhancing the power to identify stable neural features associated with individual differences in behavior.^29^

#### Connectome-based predictive modeling

CPM was applied to identify the brain features that represent individual differences in handwriting speed. This approach provides a general framework to predict behavior using brain connectivity metrics.^23^ In this study, leave-one-out cross-validation (LOOCV) was employed to evaluate the predictive performance of the model. In each iteration, one participant was considered as the test set, and the remaining participants were used as the training set. The *Z*-scores for handwriting speed were calculated in the training set, and the mean and standard deviation obtained in the training set were then used to calculate the *Z*-score for handwriting speed in the test set. This strategy ensures that the normalization of the training set is conducted independently of the test set, thereby preventing information leakage between the two sets.^23^

Feature selection was performed by linear regression analysis in the training set. The GFC that demonstrated a significant correlation with handwriting speed was retained as a feature, after controlling for age, sex and head motion. Head motion was quantified by the sum of the average FD across the two task runs and the resting-state run. The significance threshold was set at *p* < 0.001, uncorrected for multiple comparisons. Then, for each participant in the training set, we separately summed the connectivity strength (i.e., Fisher’s *Z* scores) of the GFC positively/negatively correlated with handwriting speed, yielding positive/negative summary features.

Three CPM models (positive, negative and combined) were constructed in the training set, with handwriting speed as the dependent variable of linear regression and the summary features as the independent variables. The CPM models were then applied to predict handwriting speed for the participant in the test set. The correlation coefficient between the observed and predicted scores was calculated to determine predictive accuracy.

The GFC that appeared at least 90% of the iterations was identified as a brain “fingerprint” of handwriting speed. The results were visualized using BrainNet Viewer.^59^ According to the network to which the two nodes of each GFC belonged, we divided the identified GFC into intra-network connectivity (two nodes belong to the same network) and inter-network connectivity (two nodes belong to distinct networks). The node degree is defined as the number of edges directly connected to a given node. The sum of the degrees of all nodes within a network was used to measure the weight of that network in predicting the corresponding behavior indicator.^72^

#### Internal validation of the predictive model

Several procedures were conducted to verify the reliability of the main results. First, to evaluate the impact of the significance thresholds for feature selection, the thresholds for determining the GFC that significantly correlated with handwriting speed were adjusted to *p* < 0.0025 and *p* < 0.0005, respectively. Second, given that the filtering criteria differed for task-related (high pass at 0.008 Hz) and resting-state timeseries (0.008–0.09 Hz), the GFC might be confounded by these filtering parameter differences. To examine this effect, we reran the analysis with the timeseries of tasks also band-pass filtered at 0.008–0.09 Hz, and then concatenated them with the timeseries of the resting state to calculate the GFC. Finally, to assess the influence of node location determination, we reran the analysis using the Craddock atlas, which includes 200 cortical and subcortical regions.^73^

We selected a threshold of 90% to determine and visualize reliable features within the CPM framework. This choice balances consistent feature selection across iterations with retaining sufficient features for meaningful interpretation.^23^ Furthermore, sensitivity analyses were conducted to evaluate the stability of the result across different thresholds. Specifically, we investigated the number of retained edges and the resulting functional connectivity matrices at 85%, 95%, and 100% thresholds. Spearman correlation analysis was then conducted to assess the similarity between these matrices and the one obtained at the 90% threshold.

#### External validation of the predictive model

An external validation was conducted by collecting data from a group of 24 children to verify the generalizability of the predictive model. Handwriting speed of the children was assessed using a character writing task, in which the child participants were asked to handwrite digits from 1 to 10 in Chinese as fast and legibly as possible within one minute. Handwriting speed was evaluated by the number of correctly written characters.^9^

During the fMRI scan, children performed a similar delayed copying task. The stimuli were 16 HFCs and 16 LFCs. Each participant completed two task runs. Each run consisted of two blocks of copying HFCs and two blocks of copying LFCs. Each block started with a 2-second instruction, followed by four trials. Each trial began with a centrally presented "+" symbol for 0.5 seconds, followed by a character stimulus for 1.2 seconds. After a 0.5-second blank screen, a cursor appeared for 5.3 seconds, during which participants copied the character at their usual speed. Adjacent task blocks are inserted with a 12-second rest block.

Additionally, children underwent the same resting-state scan as the adults. The fMRI preprocessing pipeline for children was identical to that for adults. The head motion of all child participants met the criterion of an average FD less than 0.5 mm.^66^ Subsequently, the same CPM procedure was used to analyze the predictive performance of children's GFC for identifying individual differences in handwriting speed, while controlling for sex, age, head motion and normal intelligence quotient (evaluated by the Combined Raven’s Progressive Matrices) during feature selection.

#### GFC model predicts reading ability

To explore whether the GFC that shared features of handwriting task-related and resting-state fMRI data could predict reading ability, we extended our analysis by using reading scores as the dependent variable in the CPM procedure. Given the complexity of handwriting processing, which involves both central processes (linguistic operations) and peripheral processes (motor execution), handwriting and reading abilities may be characterized by both shared and distinct GFC features. Therefore, whole-brain GFC was included in the CPM analysis to identify stable features that capture individual differences in reading ability.

### Quantification and statistical analysis

#### Normality test of behavioral data

For the final sample of 50 participants, Shapiro–Wilk tests were employed to examine whether the handwriting speed and reading scores conform to a normal distribution. This analysis was conducted using IBM SPSS Statistics software (version 25.0). Data were considered to follow a normal distribution when the *p*-value of the Shapiro–Wilk test is > 0.05.

#### Significance test of predictive accuracy

In the CPM analysis, a permutation test (1,000 iterations) was performed to assess the significance of the predictive accuracy (i.e., the correlation coefficient between the observed and predicted scores). During each iteration, raw scores of the behavior indicator were randomly assigned to participants, followed by implementing LOOCV. The *p* value of the permutation test was calculated as the proportion of iterations whose prediction accuracy was greater than or equal to the true prediction accuracy. This permutation test was conducted in MATLAB (version R2020a).
