# Supplementary material for: Connectome-based predictive modeling of handwriting and reading using task-evoked and resting-state functional connectivity
Source: iScience. 2025 Jul 7;28(8):113075. doi: 10.1016/j.isci.2025.113075 (PMC12335965; doi:10.1016/j.isci.2025.113075)
Supplement: Document S1. Figures S1–S6 [file mmc1.pdf]

iScience, Volume *28*

Supplemental information

Connectome-based predictive modeling of handwriting and reading using task-evoked and resting-state functional connectivity

Junjun Li, Dai Zhang, Huan Ren, Ke Zhou, and Yang Yang

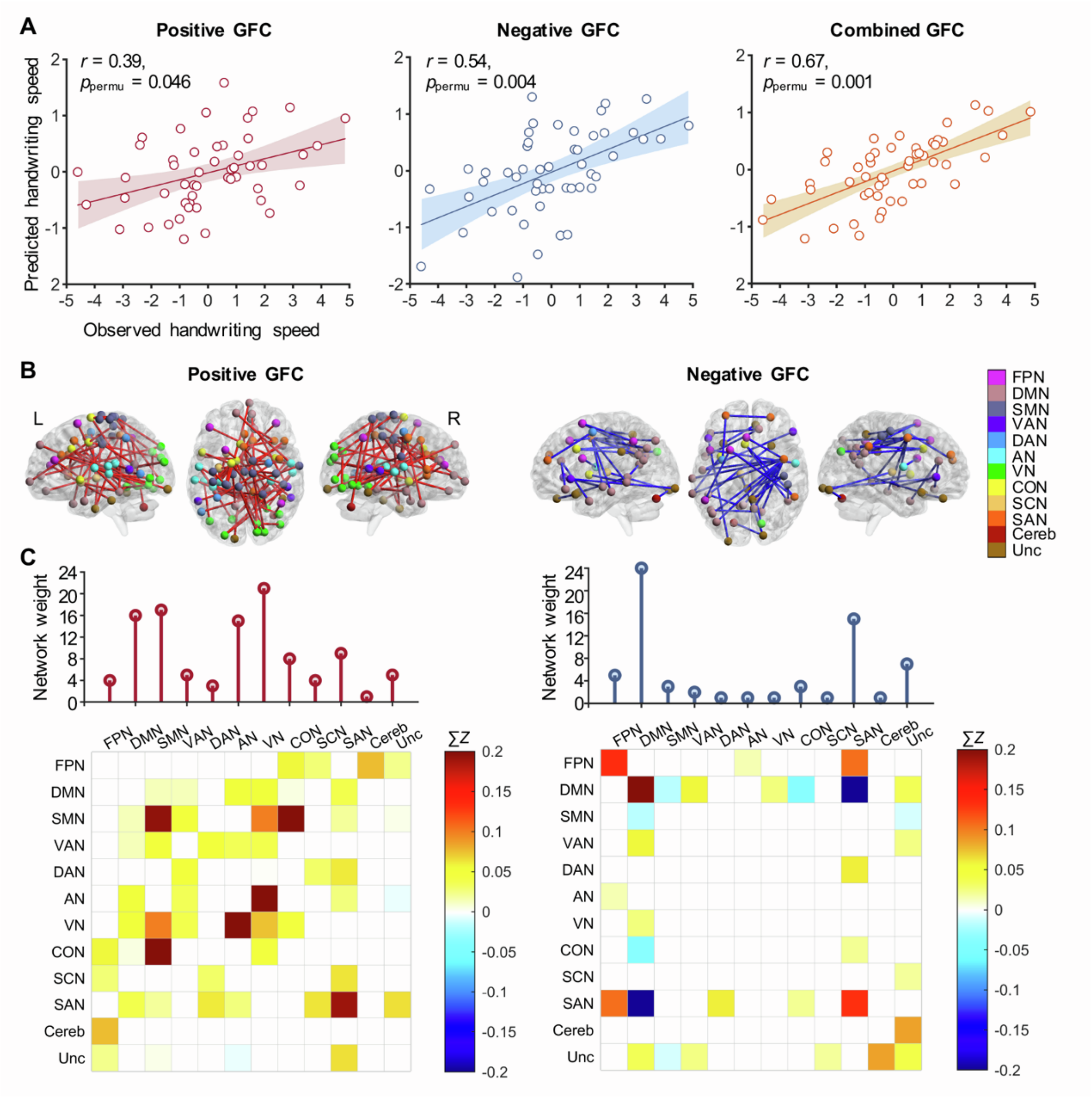

Figure S1. Internal verification results of handwriting speed predictions using a more lenient threshold ($p < 0.0025$), related to Figure 2. (A) Scatterplots showing the correlations between observed scores and predicted scores of handwriting speed based on positive, negative, and combined (positive and negative) GFC. (B) Positive and negative GFC contributing to handwriting speed predictions. The colors of the nodes indicate the networks to which they belong. (C) Network distribution of the predictive GFC and the weight of each network. Network weights are calculated as the sum of node degrees within that network. Matrix plots represent the connectivity strength between pairs of the 12 brain networks. The colorbars map the color of each matrix element to the sum of the connectivity strength (Fisher's *Z* scores) across all the edges connecting the networks. GFC, general functional connectivity; FPN, frontal-parietal network; DMN, default mode network; SMN, somatosensory motor network; VAN, ventral attention network; DAN, dorsal attention network; AN, auditory network; VN, visual network; CON, cingulo-opercular network; SCN, subcortical network; SAN, salience network; Cereb, cerebellum; Unc, uncertain; L, left; R, right. p_{permu} = the *p* value obtained by the permutation test.

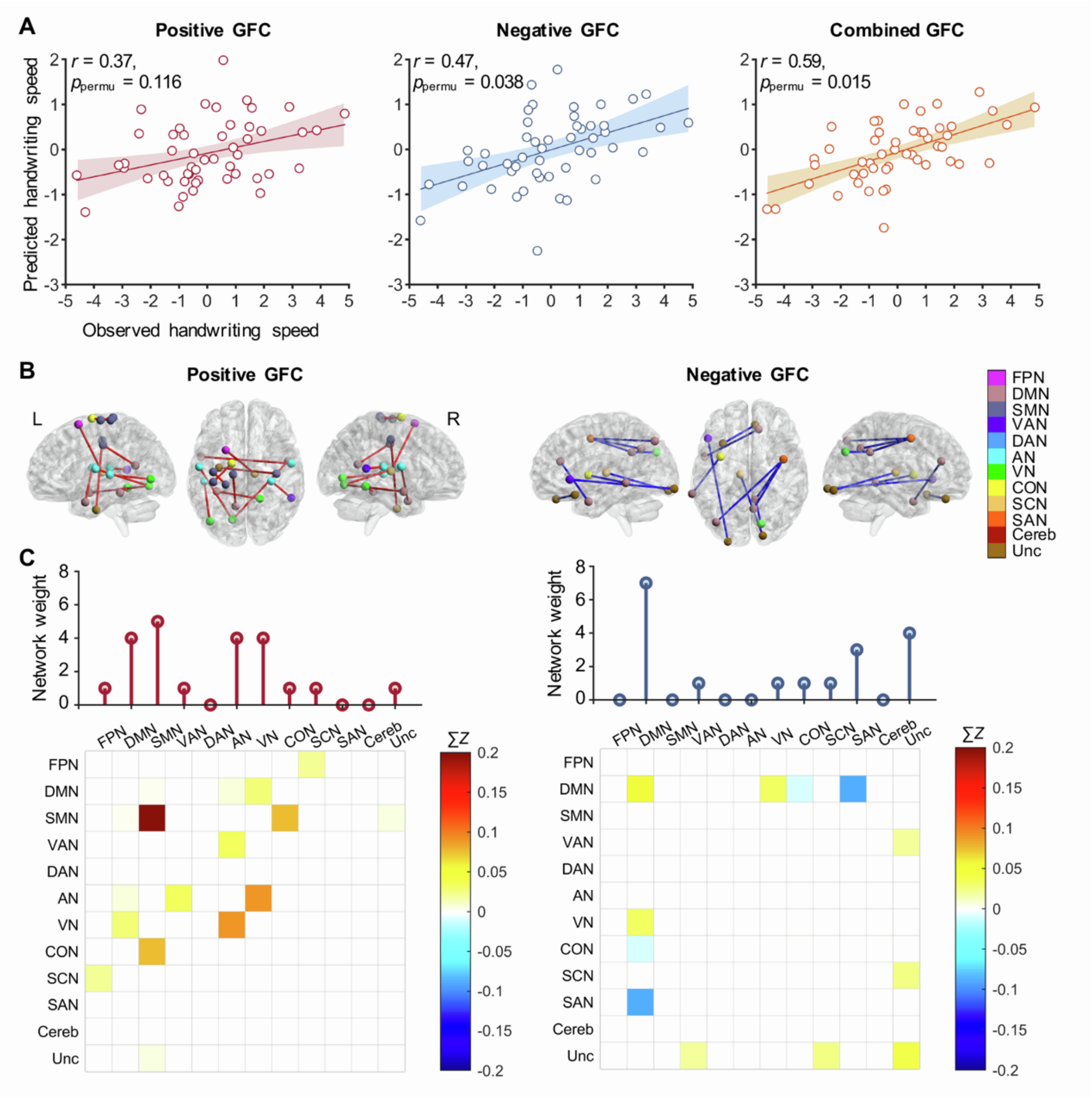

Figure S2. Internal verification results of handwriting speed predictions when a more stringent threshold (p < 0.0005) was applied, related to Figure 2. (A) Scatterplots showing the correlations between observed scores and predicted scores of handwriting speed based on positive, negative, and combined GFC. (B) Positive and negative GFC contributing to handwriting speed predictions. (C) Network distribution of the predictive GFC and the weight of each network. Network weights are calculated as the sum of node degrees within that network. Matrix plots represent the connectivity strength between pairs of the 12 brain networks. The colorbars map the color of each matrix element to the sum of the connectivity strength (Fisher's Z scores) across all the edges connecting the networks. GFC, general functional connectivity; FPN, frontal-parietal network; DMN, default mode network; SMN, somatosensory motor network; VAN, ventral attention network; DAN, dorsal attention network; AN, auditory network; VN, visual network; CON, cingulo-opercular network; SCN, subcortical network; SAN, salience network; Cereb, cerebellum; Unc, uncertain; L, left; R, right. p_{permu} = the p value obtained by the permutation test.

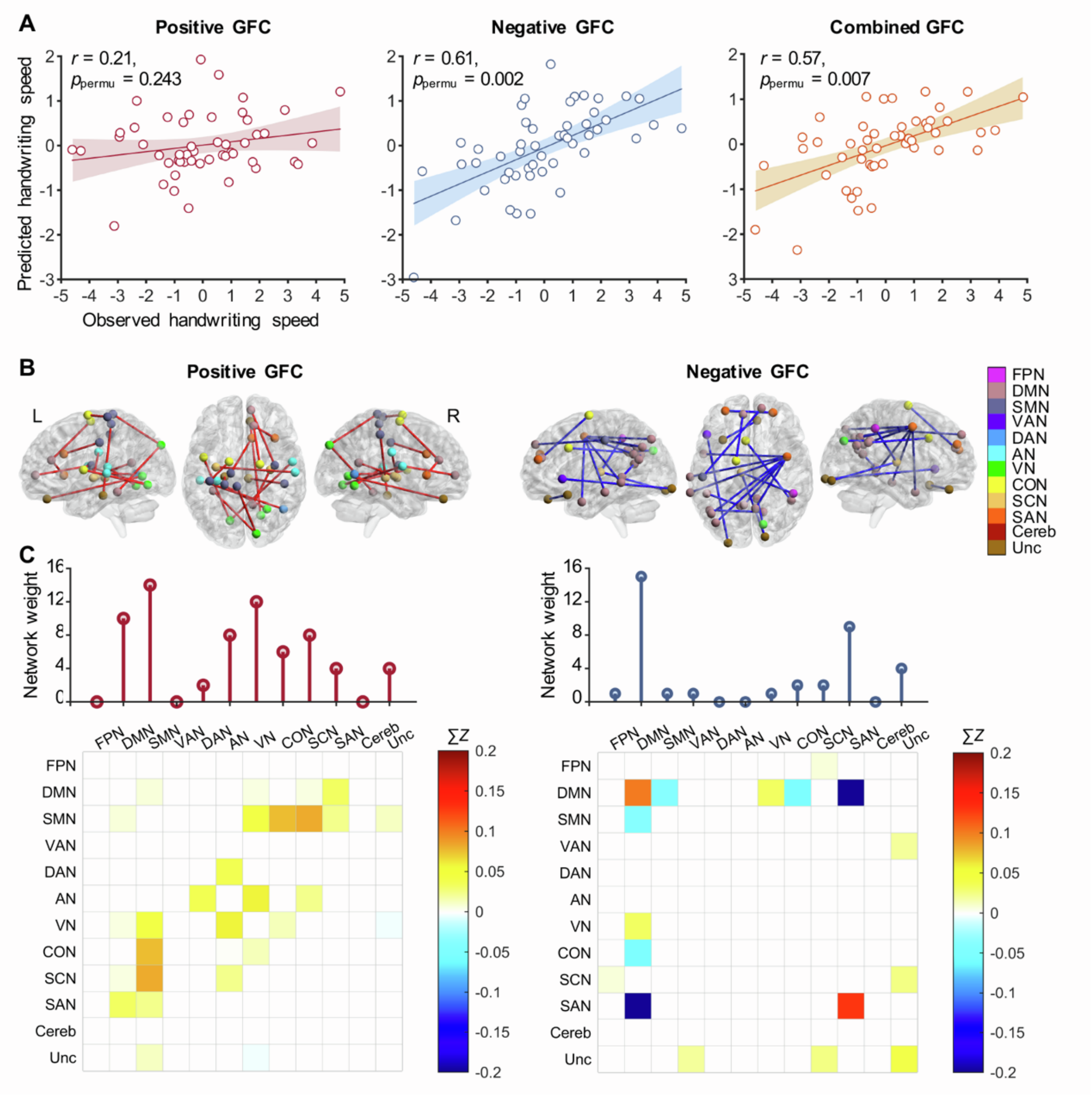

Figure S3. Internal verification results of handwriting speed predictions when both the task and the resting-state time series were band-pass filtered at 0.008-0.009 Hz, related to Figure 2. (A) Scatterplots showing the correlations between observed scores and predicted scores of handwriting speed based on positive, negative, and combined GFC. (B) Positive and negative GFC contributing to handwriting speed predictions. (C) Network distribution of the predictive GFC and the weight of each network. Network weights are calculated as the sum of node degrees within that network. Matrix plots represent the connectivity strength between pairs of the 12 brain networks. The colorbars map the color of each matrix element to the sum of the connectivity strength (Fisher's *Z* scores) across all the edges connecting the networks. GFC, general functional connectivity; FPN, frontal-parietal network; DMN, default mode network; SMN, somatosensory motor network; VAN, ventral attention network; DAN, dorsal attention network; AN, auditory network; VN, visual network; CON, cingulo-opercular network; SCN, subcortical network; SAN, salience network; Cereb, cerebellum; Unc, uncertain; L, left; R, right. p_{permu} = the *p* value obtained by the permutation test.

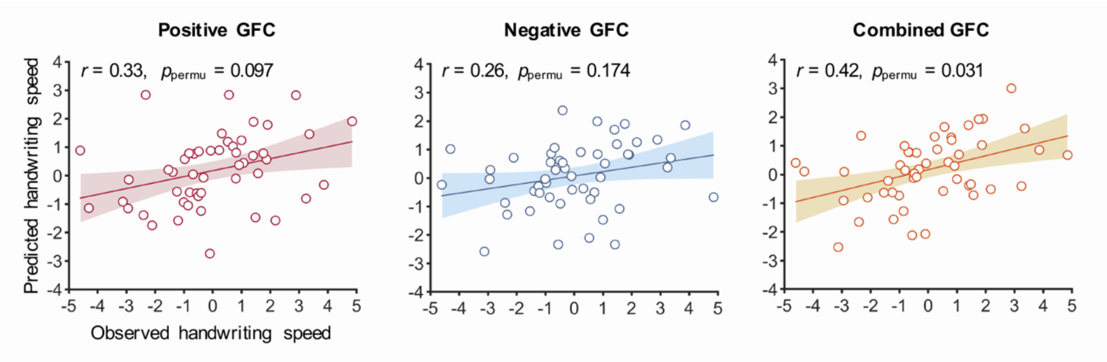

Figure S4. Verification results of handwriting speed predictions when the Craddock atlas was applied, related to Figure 2. Scatterplots show the correlations between observed scores and predicted scores of handwriting speed based on the positive, negative, and combined (positive and negative) GFC. GFC, general functional connectivity. p_{permu} = the p value obtained by the permutation test.

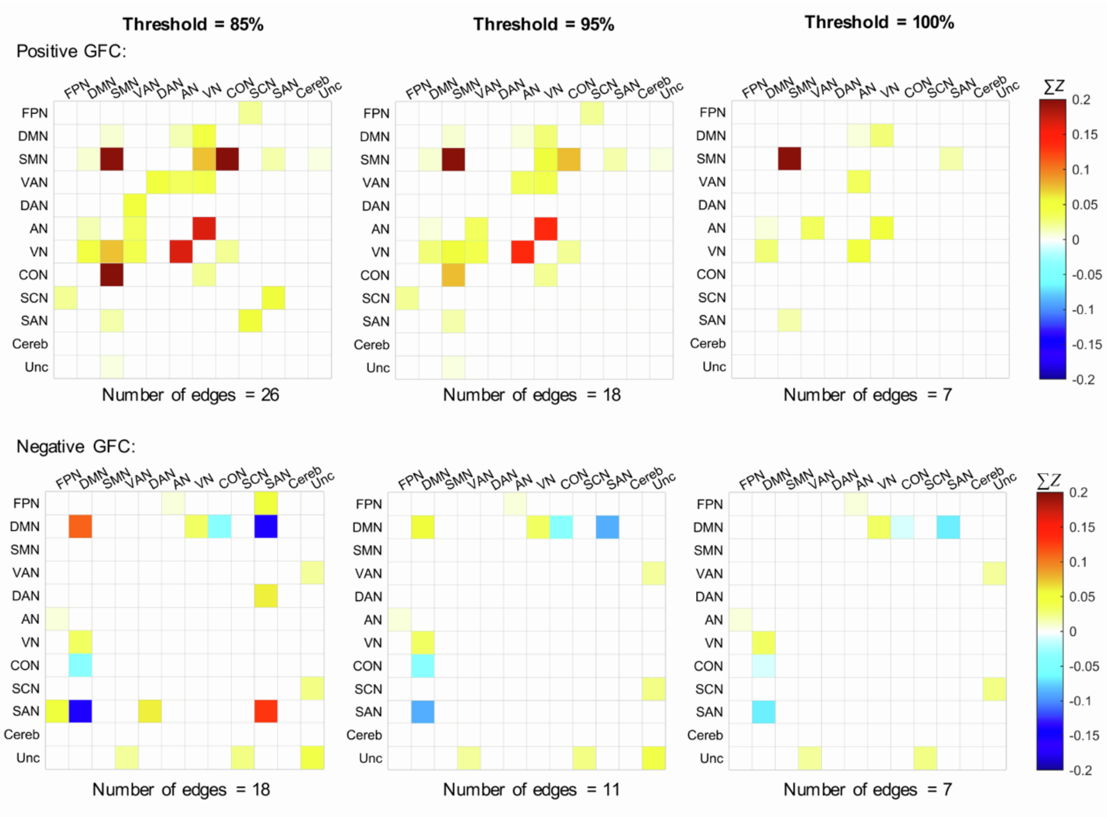

Figure S5. Network distribution of positive and negative GFC when different thresholds were applied to identify GFC stably related to handwriting speed across the CPM cross-validation procedure, related to Figure 2. Matrix plots represent the connectivity strength between pairs of the 12 brain networks. The colorbars map the color of each matrix element to the sum of the connectivity strength (Fisher's Z scores) across all the edges connecting the networks. GFC, general functional connectivity; FPN, frontal-parietal network; DMN, default mode network; SMN, somatosensory motor network; VAN, ventral attention network; DAN, dorsal attention network; AN, auditory network; VN, visual network; CON, cingulo-opercular network; SCN, subcortical network; SAN, salience network; Cereb, cerebellum; Unc, uncertain.

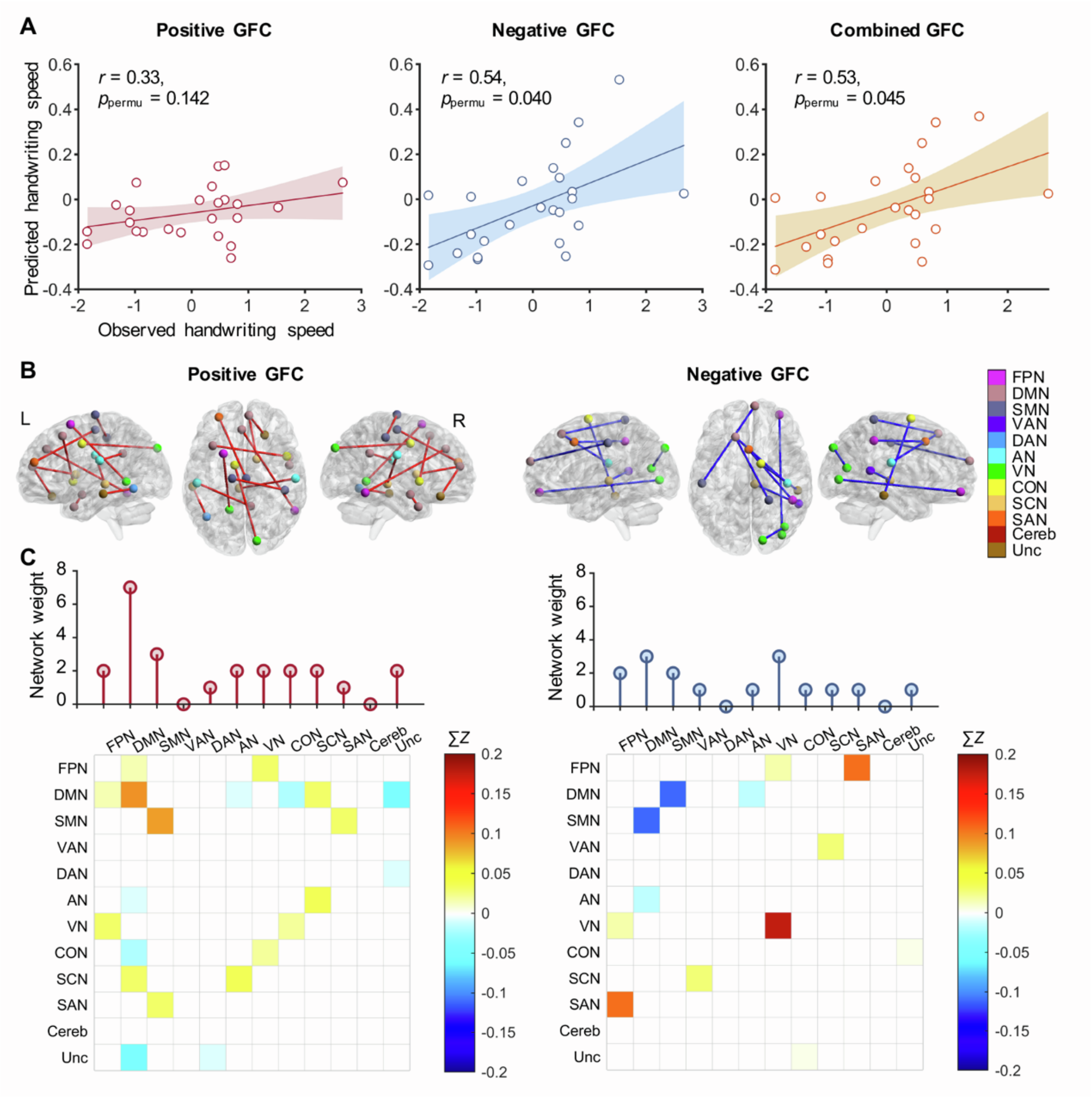

Figure S6. External verification results of handwriting speed predictions, related to Figure 2. (A) Scatterplots showing the correlations between observed scores and predicted scores of handwriting speed based on positive, negative, and combined GFC. (B) Positive and negative GFC contributing to handwriting speed predictions. (C) Network distribution of the predictive GFC and the weight of each network. Network weights are calculated as the sum of node degrees within that network. Matrix plots represent the connectivity strength between pairs of the 12 brain networks. The colorbars map the color of each matrix element to the sum of the connectivity strength (Fisher's *Z* scores) across all the edges connecting the networks. GFC, general functional connectivity; FPN, frontal-parietal network; DMN, default mode network; SMN, somatosensory motor network; VAN, ventral attention network; DAN, dorsal attention network; AN, auditory network; VN, visual network; CON, cingulo-opercular network; SCN, subcortical network; SAN, salience network; Cereb, cerebellum; Unc, uncertain; L, left; R, right. p_{permu} = the *p* value obtained by the permutation test.
